# Factors associated with PrEP‐era HIV seroconversion in a 4‐year U.S. national cohort of *n* = 6059 sexual and gender minority individuals who have sex with men, 2017−2022

**DOI:** 10.1002/jia2.26312

**Published:** 2024-06-25

**Authors:** Christian Grov, Yan Guo, Drew A. Westmoreland, Alexa B. D'Angelo, Chloe Mirzayi, Michelle Dearolf, Pedro Carneiro, Meredith Ray, David Pantalone, Adam W. Carrico, Viraj V. Patel, Sarit A. Golub, Sabina Hirshfield, Donald R. Hoover, Denis Nash

**Affiliations:** ^1^ City University of New York (CUNY) Graduate School of Public Health and Health Policy New York City New York USA; ^2^ CUNY Institute for Implementation Science in Population Health New York City New York USA; ^3^ University of Florida Gainesville Florida USA; ^4^ University of Memphis Memphis Tennessee USA; ^5^ University of Massachusetts Boston Boston Massachusetts USA; ^6^ Florida International University Miami Florida USA; ^7^ Albert Einstein College of Medicine Bronx New York USA; ^8^ Hunter College of CUNY New York City New York USA; ^9^ State University of New York (SUNY) Downstate Brooklyn New York USA; ^10^ Rutgers University New Brunswick New Jersey USA

**Keywords:** sexual and gender minority individuals, HIV seroconversion, pre‐exposure prophylaxis, methamphetamine, race and ethnicity, socio‐economic status

## Abstract

**Introduction:**

Community‐based cohort studies of HIV seroconversion can identify important avenues for enhancing HIV prevention efforts in the era of pre‐exposure prophylaxis (PrEP). Within individuals, one can assess exposure and outcome variables repeatedly and with increased certainty regarding temporal ordering. This cohort study examined the association of several risk factors with subsequent HIV seroconversion.

**Methods:**

We report data from a 4‐year study (2017−2022) of 6059 HIV seronegative sexual and gender minority individuals who have sex with men who had indications for‐, but were not using‐, PrEP at enrolment. Participants completed repeat exposure assessments and self‐collection of biospecimens for HIV testing. We examined the roles of race and ethnicity, socio‐economic status, methamphetamine use and PrEP uptake over the course of follow‐up in relation to HIV seroconversion.

**Results:**

Over 4 years, 303 of the participants seroconverted across 18,421 person‐years (incidence rate = 1.64 [95% CI: 1.59−1.70] per 100 person‐years). In multivariable discrete‐time survival analysis, factors independently associated with elevated HIV seroconversion risk included being Black/African American (adjusted risk ratio [aRR]: 2.44, 1.79−3.28), Hispanic/Latinx (1.53, 1.19−1.96), housing instability (1.58, 1.22−2.05) and past year methamphetamine use (3.82, 2.74−5.33). Conversely, time since study enrolment (24 vs. 12 months, 0.67, 0.51−0.87; 36 months, 0.60, 0.45−0.80; 48 months, 0.48, 0.35−0.66) and higher education (master's degree or higher vs. less than or equal to high school, 0.36, 0.17−0.66) were associated with reduced seroconversion risk. Compared to non‐PrEP users in the past 2 years without a current clinical indication, those who started PrEP but then discontinued had higher seroconversion risk, irrespective of clinical indication (3.23, 1.74−6.46) or lack thereof (4.30, 1.85−9.88). However, those who initiated PrEP in the past year (0.14, 0.04−0.39) or persistently used PrEP in the past 2 years (0.33, 0.14−0.74) had a lower risk of seroconversion. Of all HIV seroconversions observed during follow‐up assessments (12, 24, 36 and 48 months), methamphetamine was reported in the 12 months *prior* 128 (42.2%) times (overall).

**Conclusions:**

Interventions that acknowledge race and ethnicity, economic variables such as education and housing instability, and methamphetamine use are critically needed. Not only are interventions to engage individuals in PrEP care needed, but those that retain them, and re‐engage those who may fall out of care are essential, given the exceptionally high risk of seroconversion in these groups.

## 1 INTRODUCTION

Sexual minority individuals represent 2−5% of the United States, yet account for more than two‐thirds of new HIV acquisitions [1−4]. Meanwhile, gender minority individuals (transgender [trans] men and trans women) accounted for 2% of new HIV acquisitions in 2019, while representing ∼1% of the United States [5−7].

A complex set of factors have hindered our ability to reduce HIV incidence among sexual and gender minority (SGM) individuals who have sex with men. HIV pre‐exposure prophylaxis (PrEP) has been food and drug administration (FDA)‐approved for more than a decade. However, we have observed slow adoption of PrEP and seen racial/ethnic disparities in PrEP uptake—with White SGM adopting PrEP at higher rates than persons of colour [8, 9].

Meanwhile, maintenance of PrEP use is challenging, because PrEP delivery is a complex form of healthcare—regular clinical appointments, bloodwork and pharmacy coordination [10]. Maintenance of PrEP requires continued access to healthcare through, for example, job stability that provides health insurance, as well as reliable transportation for PrEP‐related appointments. Economic precarity can affect access to food and/or stable housing undermining individuals’ PrEP use and maintenance [11−13], as the need to secure fundamental necessities usurp individuals’ ability to move through the PrEP cascade [14].

Meanwhile, substance use, specifically methamphetamine (meth), can lead to hazardous negative outcomes including HIV acquisition. It is well documented that substance rates use are higher among SGM populations. Reasons are complex and include the use of substances for pleasure and escapism from adverse social‐/societal‐/political‐stigmatization of SGM populations (internalized homophobia, minority stress) [9]. Meth use estimates ranging from 7.4% to 30%. Findings from our team and others [15, 16] demonstrate that meth use is disproportionately impacting SGM individuals from marginalized racial and ethnic groups, and account for one‐in‐three new HIV acquisitions among SGM individuals [17]. Methamphetamine increases sexual libido while simultaneously decreasing inhibitions—creating a behavioural pathway for HIV transmission. Additionally, methamphetamine has the potential to interrupt effective PrEP treatment/care, leading to missed doses or failure to remain on PrEP longitudinally. Severe methamphetamine use disorders could disrupt other facets of daily life, leading to job loss and negative socio‐economic outcomes.

Large, community‐based cohort studies of HIV seroconversion can identify important avenues for enhancing HIV prevention by examining factors associated with HIV seroconversion in geographically and racially/ethnically diverse populations. In doing so, we have the potential to generate information that could inform interventions to improve the delivery and impact of HIV‐prevention strategies [18]. This study reports on data from a 4‐year (2017–2022) prospective cohort study of 6059 HIV‐vulnerable seronegative SGM who have sex with men who were clinically indicated for‐, but not using‐, PrEP at enrolment.

## 2 METHODS

### 2.1 Population and procedures

*Together 5000* (T5K) was a U.S. national cohort study to investigate missed opportunities for HIV prevention among cisgender (cis) men and trans individuals who have sex with men. Participants were recruited in 2017−2018 through geosocial applications. Inclusion criteria were: (1) identifying as a cis man or trans person; (2) being aged 16−49; (3) having had at least two male sex partners in the past 3 months; (4) not reporting being on PrEP; (5) not participating in an HIV vaccine or PrEP clinical trial; (6) residing in the United States or its territories; (7) reporting HIV negative or unknown HIV status; and (8) exhibiting behaviours indicative of clinical indication for PrEP [19]. Additional details have been published elsewhere [19, 20]. Participants provided informed consent and all procedures were approved by the Institutional Review Board.

**Figure 1:**
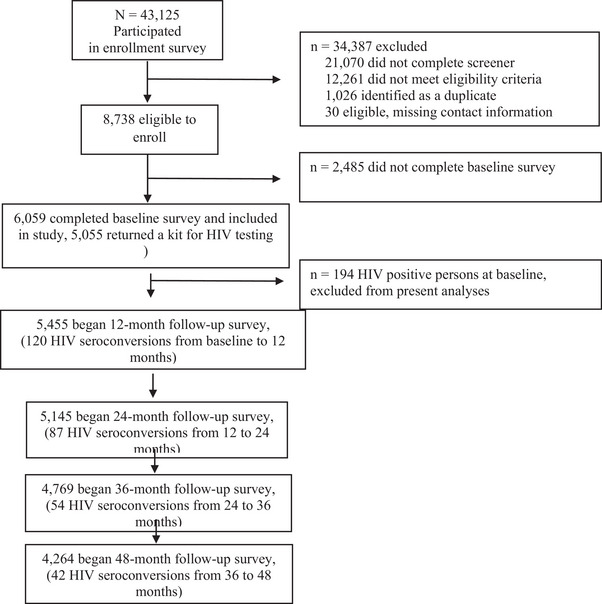
Flow diagram of *Together 5000* cohort inclusion and follow‐up.

Out of the initial 43,125 participants for the screening survey, 34,387 were excluded due to ineligibility or missing key information, resulting in 8738 who were eligible to enrol (Figure 1). Anyone who tested HIV positive at enrolment (i.e. prevalent cases) were excluded from the present analyses. Of these, 6059 completed a baseline survey and comprise the sample (denominator) for the present analyses.

At annual assessments, participants completed an online survey and were mailed an at‐home HIV specimen collection kit. However, those having tested HIV positive at a prior assessment or self‐reporting an HIV seroconversion *between* assessments were not asked to test again. Those reporting an HIV seroconversion *between* assessments were asked to provide photographic proof (e.g. an HIV medication bottle with name/date visible). Similarly, those who reported being on PrEP at a follow‐up assessment were asked to provide a photo of their PrEP bottle with name and date visible in lieu of testing—as they would be receiving regular testing from their care provider. A digital photo of their current PrEP prescription bottle served as a proxy for recent HIV testing. However, if a person reported having *discontinued* PrEP, they were asked to test with us again. Incentives ranged between $15−$25 for completing a survey, and $15−$25 for returning an HIV test kit, or for providing photographic proof.

All participants were provided with information on PrEP, where they could get access to it, as well as HIV testing and other forms of HIV prevention. Participants who tested HIV positive were provided tailored resources for linkage to care.

Completion rates were high among those who started surveys—96.1% (5243 participants), 97.6% (5024), 98.3% (4688) and 97.7% (4168) completing the 12‐, 24‐, 36‐ and 48‐month assessments, respectively. Our analysis included participants who *began* the annual surveys.

### 2.2 HIV seroconversion

The primary outcome was incident HIV acquisitions as measured by a positive HIV serologic test following the negative HIV serologic test performed at enrolment. The timing of HIV seroconversion was determined by the year of assessment. For most of the study, we used the OraSure HIV‐1 specimen collection device [27]. Participants used prepaid shipping materials to send their oral fluid samples to a lab for analysis. The COVID‐19 pandemic disrupted some study procedures, resulting in a pause in mailing test kits between March and June 2020. In June, we resumed testing for all participants overdue to test using dried blood spots instead of oral fluid specimens (1096 valid samples were received by the lab, 15 were HIV reactive). That November, we resumed testing with the OraSure HIV‐1 specimen collection device for the remainder of the study.

### 2.3 Predictors of HIV seroconversion

We examined potential factors associated with HIV seroconversion, including demographic characteristics (age, race/ethnicity), socio‐economic status (education, food insecurity, housing instability) and behaviour (prior year methamphetamine use, PrEP use patterns in the past 2 years). All variables, except race/ethnicity, were time‐varying and only those values temporally preceding seroconversion were used in analyses.

Race and ethnicity was grouped as non‐Hispanic White, non‐Hispanic Black, Hispanic/Latinx and “other.” Education was categorized into four groups: ≤ high school, some college, bachelor's degree, master's degree or higher. We assessed food insecurity using the U.S. Department of Agriculture's 6‐item survey starting at the 12‐month assessment and operationalized this by combining the low and very low categories for analysis [21]. We assessed housing instability by asking participants if they had experienced situations such as living in a car, staying in a shelter, couch surfing or being homeless in the past year (1 = yes, 0 = no). Both food insecurity and housing instability served as proxies for socio‐economic status, capturing essential needs more accurately than variables like income alone.

We incorporated data on prior year methamphetamine use, as well as its one‐time‐lagged term (methamphetamine use reported in the survey of the year before).

To assess its relationship with HIV seroconversion, we also examined PrEP use patterns over 2 years, accounting for its prior year use, one‐time‐lagged term and clinical indication. PrEP use was assessed annually, with participants indicating whether they took PrEP the previous year. Annual PrEP indications aligned closely with the Centers for Disease Control and Prevention's 2017 guidelines [22]. We divided PrEP use patterns into six mutually exclusive categories: (1) no PrEP use in the past 2 years, not currently clinically indicated; (2) no PrEP use in the past 2 years, but currently clinically indicated; (3) initiated PrEP in the past year, regardless of indication; (4) discontinued PrEP without a current clinical indication; (5) discontinued PrEP with a current clinical indication; and (6) maintained continuous PrEP use in the past 2 years, regardless of indication.

### 2.4 Statistical analysis

First, we described baseline characteristics (frequencies, percentages, median, interquartile range [IQR]). Then, we calculated HIV incidence rates per 100 person‐years by PrEP and compared rates between participants who initiated PrEP and those who did not during the study using Poisson regression reporting rates, rate ratios and 95% confidence intervals.

To investigate time‐varying and time‐invariant factors associated with HIV seroconversion, we used discrete‐time survival analysis, suitable for discretely measured time events [23]. To account for the nonlinear effect of age, we used natural splines rather than dividing age into distinct categories. We employed R's default settings for natural splines, which typically include three internal knots positioned at the quartiles. To mitigate bias from attrition, we calculated cohort attrition weights for each assessment time point based on survey response and test kit return then used them in our multivariable analysis [24, 25]. These “attrition weights” balance disproportionate groups of participants that were unintentionally over‐ or under‐represented during the study (e.g. lost to follow‐up) [25, 26]. Weights were defined as the inverse probability of completing a survey (or returning a test kit), and modelled using logistic regression given participant age, gender, sexual orientation, race, employment status, education, income, health insurance status, housing instability and engagement in transactional sex [27]. To minimize missing data when calculating the attrition weights, we “back‐filled” using participants’ most recent data. Extreme weights (>95%) were trimmed to the 95th percentile value.

Our final observation‐weighted multivariable discrete‐time survival analysis included time points, age, race/ethnicity, education, housing instability, past year methamphetamine use, its one‐time‐lagged term and PrEP use patterns in the past 2 years. We reported risk ratios (RR) and 95% CIs. To ensure the robustness of estimates, we compared results from this weighted model with those from the unweighted model, a model including only significant factors (excluding factors not significant in the multivariable model), and a model where age was treated as a categorical variable. Our analyses used the GLM function in R 4.2.2 with a binomial distribution and clogclog link (R Core Team, Vienna, Austria).

## 3 RESULTS

### 3.1 Demographics

Table 1 presents the characteristics of the 6059 participants. The median age was 29.0 years (IQR: 25.0−36.0), with 52.3% White, 10.7% Black/African American, 24.5% Hispanic/Latinx and 12.5% from other racial/ethnic groups. Among individuals who seroconverted during follow‐up, a higher proportion were Black/African American (15.5%) and Hispanic/Latinx (29.7%) compared to those who did not seroconvert during follow‐up, where the proportions were 10.4% for Black/African American and 24.2% for Hispanic/Latinx individuals. Additionally, 38.2% of all participants had at least a bachelor's degree. Most participants were cisgender males (97.5%). The proportions are almost the same by HIV seroconversion. However, educational attainment was lower among those who seroconverted, with only 24.5% holding a bachelor's degree or higher, compared to 39.3% who did not seroconvert. Concerning socio‐economic challenges, 27.6% of the participants experienced food insecurity and 29.8% faced housing instability over the past year. The rates of food insecurity (40.9%) and housing instability (39.9%) were significantly higher among individuals who seroconverted, compared to 27.0% and 29.3%, respectively, for those who did not seroconvert. Similarly, a higher proportion of participants used methamphetamine (46.9%) among those who seroconverted than among those who did not seroconvert (21.0%).

**Table 1:** Characteristics of sexual and gender minority individuals in a U.S.‐based longitudinal cohort, *Together 5000*, 2017−2022

	*n* (%) or median (IQR)		
Characteristic	All participants	Participants who seroconverted	Participants who did not seroconvert
Total number	6059 (100%)	303 (5.0%)	5756 (95.0%)
Age	29.0 (25.0−36.0)	30.0 (25.0−36.0)	29.0 (25.0−36.0)
Race/ethnicity			
Non‐Hispanic White	3167 (52.3%)	135 (44.6%)	3032 (52.7%)
Non‐Hispanic Black	648 (10.7%)	47 (15.5%)	601 (10.4%)
Hispanic/Latinx	1485 (24.5%)	90 (29.7%)	1395 (24.2%)
Other	759 (12.5%)	31 (10.2%)	728 (12.6%)
Gender			
Cisgender male	5909 (97.5%)	295 (97.4)	5614 (97.5%)
Other	150 (2.5%)	8 (2.6)	142 (2.5%)
Education			
≤ High school	997 (16.5%)	59 (19.5)	938 (16.3%)
Some college	2725 (45.0%)	170 (56.1)	2555 (44.4%)
Bachelor's degree	1616 (26.7%)	65 (21.5%)	1551 (26.9%)
Master's degree or higher	721 (11.9%)	9 (3.0%)	712 (12.4%)
Food insecurity (at 12 months)^a^			
No	3055 (50.4%)	134 (44.2%)	2921 (50.7%)
Yes	1674 (27.6%)	187 (40.9%)	1550 (27.0%)
Missing values	1330 (22.0%)	45 (14.9%)	1285 (22.3%)
Had unstable housing	1808 (29.8%)	121 (39.9%)	1687 (29.3%)
Used methamphetamine in the past	1351 (22.3%)	142 (46.9%)	1209 (21.0%)

^a^ Questions on food insecurity were not included at baseline, we utilized data from the 12‐month assessment as the baseline for food insecurity. IQR refers to interquartile range.

### 3.2 HIV incidence

In total, 5455/6059 (90.0%) began the 12‐month assessment, 5145/6059 (84.9%) the 24‐month, 4769/6059 (78.7%) the 36‐month and 4264/6059 (70.4%) the 48‐month assessment. At 12 months, *n* = 120 new HIV seroconversions were identified (62 of 3445 samples received tested positive, 58 reported a positive test between assessments). At 24 months, *n* = 87 additional HIV seroconversions were identified (36 of 2753 samples received tested positive, 51 reported a positive test between assessments). At 36 months, *n* = 54 new HIV seroconversions were identified (29 of 2586 samples received tested positive, 25 reported a positive test between assessments). And at 48 months, *n* = 42 new HIV seroconversions were identified (17 of 2308 samples received tested positive, 25 reported a positive test between assessments).

Table 2 shows HIV incidence based on PrEP use. Over 4 years, 303 of the participants HIV seroconverted during 18,421 person‐years of follow‐up, resulting in an incidence rate of 1.64 (95% CI 1.59−1.70) cases per 100 person‐years. Among those who initiated PrEP, there were 20 seroconversions over 4872 person‐years, an incidence rate of 0.41 (0.35−0.47) per 100 person‐years. Conversely, among those who did not initiate PrEP during the study, there were 283 seroconversions over 13,549 person‐years, a rate of 2.09 (2.06−2.11) per 100 person‐years. The crude incidence rate ratio was 5.10 (3.24−8.02; *p*<0.001) for those who did not initiate PrEP compared to those who initiated PrEP.

**Table 2:** HIV incidence rates by PrEP usage

	Person‐years	Number of people who seroconverted	Incidence per 100 person‐years (95% CI)	Incidence rate ratio (95% CI)	*p* value
Overall	18,421	303	1.64 (1.59−1.70)	··	··
Initiated PrEP	4872	20	0.41 (0.35−0.47)	0.20 (0.13−0.31)	<0.001
Did not initiate PrEP	13,549	283	2.09 (2.06−2.11)	5.10 (3.24−8.02)	<0.001

*Note*: CI refers to confidence interval and PrEP to pre‐exposure prophylaxis.

### 3.3 Bivariate discrete‐time survival analysis

Table 3 reports the 4‐year bivariate results of discrete‐time survival analysis for factors associated with HIV seroconversion among participants clinically indicated but not using PrEP at enrolment. Factors associated with a higher risk of HIV seroconversion included being non‐Hispanic Black (risk ratio [RR]: 1.81, 95% CI 1.28−2.52), Hispanic/Latinx (RR: 1.48, 1.12−1.93), prior year food insecurity (RR: 2.24, 1.77−2.84), prior year housing instability (RR: 3.92, 3.02−5.04), past year methamphetamine use (RR: 6.23, 4.93−7.87) and its one‐time lagged term (RR: 4.43, 3.46−5.67). Conversely, time since enrolment (24 vs. 12 months, RR: 0.85, 0.40−0.67; 36 months, RR: 0.58, 0.26−0.48; 48 months, RR: 0.43, 0.19−0.37) and higher education (bachelor's degree vs. ≤ high school, RR: 0.57, 0.40−0.82; master's degree or higher, RR: 0.15, 0.07−0.30) were associated with a lower risk of seroconversion. When comparing prior 2‐year PrEP use patterns to those not using PrEP and without a current clinical indication, individuals who were either not on PrEP but clinically indicated (RR: 1.79, 1.12−3.10) or who discontinued PrEP with a clinical indication (RR: 3.41, 1.89−6.39) had a higher risk of seroconversion. In contrast, initiating PrEP (RR: 0.12, 0.03−0.37) or persistently using it (RR: 0.20, 0.08−0.46) greatly reduced this risk. The risk for those who discontinued PrEP, without a current clinical indication, was not significantly different from those who did not initiate PrEP without a clinical indication. Age and gender were not associated with HIV seroconversion.

**Table 3:** Bivariate discrete‐time survival analysis results of factors associated with HIV seroconversion among sexual and gender minority individuals in a U.S.‐based longitudinal cohort, *Together 5000*, 2017−2022

Predictors	Risk ratio	95% CI	*p* value
Other follow‐ups versus 12 months			
Time (24 months)	0.85	0.40−0.67	**<0.001**
Time (36 months)	0.58	0.26−0.48	**<0.001**
Time (48 months)	0.43	0.19−0.37	**<0.001**
Age (in years)	0.78	0.40−1.49	0.452
Cisgender male versus other	0.85	0.52−1.52	0.545
Racial/ethnic minorities versus non‐Hispanic White			
Non‐Hispanic Black	1.81	1.28−2.52	**0.001**
Hispanic/Latinx	1.48	1.12−1.93	**0.005**
Other	1.02	0.68−1.49	0.919
Higher education versus ≤ high school			
Some college	0.94	0.70−1.27	0.671
Bachelor's degree	0.57	0.40−0.82	**0.002**
Master's degree or higher	0.15	0.07−0.30	**<0.001**
Food insecure versus food secure	2.24	1.77−2.84	**<0.001**
Unstable housing versus stable housing	3.92	3.02−5.04	**<0.001**
Meth use versus non‐meth use in the past year	6.23	4.93−7.87	**<0.001**
Meth use versus non‐meth use 1−2 years ago	4.43	3.46−5.67	**<0.001**
PrEP use patterns in the past 2 years versus no PrEP use in the past 2 years, not clinically indicated			
No PrEP use, clinically indicated	1.79	1.12−3.10	**0.024**
Initiated PrEP this year	0.12	0.03−0.37	**0.001**
Quit PrEP, not clinically indicated	2.24	0.91−5.09	0.063
Quit PrEP, clinically indicated	3.41	1.89−6.39	**<0.001**
Persistently used PrEP	0.20	0.08−0.46	**<0.001**

*Note*: CI refers to confidence interval and PrEP to pre‐exposure prophylaxis. The results were not weighted.

Bold values are indicates statistically significant.

### 3.4 Multivariable results of predictors of HIV seroconversion

Table 4 presents an observation‐weighted multivariable discrete‐time survival analysis with 16,700 person‐years of follow‐up. Multivariable results mirrored bivariate findings, except for PrEP use patterns, food insecurity and one‐time lagged methamphetamine use in the past year—the latter two becoming non‐significantly associated with seroconversion risk. Factors independently associated with higher HIV seroconversion risk included being Black/African American (adjusted risk ratio [aRR]: 2.44, 1.79−3.28), Hispanic/Latinx (aRR: 1.53, 1.19−1.96), housing instability (aRR: 1.58, 1.22−2.05) and past year methamphetamine use (aRR: 3.82, 2.74−5.33). Of the *n* = 303 HIV seroconversions observed during follow‐up assessments (12, 24, 36 and 48 months), methamphetamine was reported in the 12 months *prior* 128 (42.2%) times (overall). Respectively, when examining HIV seroconversions observed at 12, 24, 36 and 48 months, 54 (45.0%), 35 (40.2%), 22 (40.7%) and 17 (40.5%) reported using methamphetamine in the year proceeding HIV seroconversion.

**Table 4:** Multivariable, observation‐weighted discrete‐time survival analysis results for HIV seroconversion among sexual and gender minority individuals in a U.S.‐based longitudinal cohort, *Together 5000*, 2017−2022

Predictors	Adjusted risk ratio	95% CI	*p* value
Other follow‐ups versus 12 months			
Time (24 months)	0.67	0.51−0.87	**0.004**
Time (36 months)	0.60	0.45−0.80	**0.001**
Time (48 months)	0.48	0.35−0.66	**<0.001**
ns(age)	0.73	0.37−1.42	0.361
Racial/ethnic minorities versus non‐Hispanic White			
Non‐Hispanic Black	2.44	1.79−3.28	**<0.001**
Hispanic/Latinx	1.53	1.19−1.96	**0.001**
Other	1.11	0.77−1.58	0.562
Cisgender male versus other	0.91	0.54−1.69	0.736
Higher education versus ≤ high school			
Some college	1.04	0.80−1.36	0.776
Bachelor's degree	0.87	0.62−1.22	0.416
Master's degree or higher	0.36	0.17−0.66	**0.002**
Food insecure versus food secure	1.01	0.80−1.26	0.966
Unstable housing versus stable housing	1.58	1.22−2.05	**0.001**
Meth use versus non‐meth use in the past year	3.82	2.74−5.33	**<0.001**
Meth use versus non‐meth use 1−2 years ago	1.38	0.99−1.94	0.060
PrEP use patterns in the past 2 years versus no PrEP and not indicated for PrEP in the past 2 years			
No PrEP and indicated for PrEP	1.69	0.98−3.22	0.079
Initiated PrEP this year	0.14	0.04−0.39	**0.001**
Quit PrEP, not indicated for PrEP	4.30	1.85−9.88	**0.001**
Quit PrEP, indicated for PrEP	3.23	1.74−6.46	**<0.001**
Persistent PrEP use	0.33	0.14−0.74	**0.007**

*Note*: CI refers to confidence interval; ns refers to natural spline; and PrEP to pre‐exposure prophylaxis. 16,700 person‐years were included in the analysis. The results were weighted with inverse probability of censoring weighting. Food insecurity variable which was significant in bivariate analysis but not in multivariable analysis was included in this model. Gender, not statistically significant in bivariate analysis, was also included in the model.

Bold values are indicates statistically significant.

Conversely, time since enrolment (24 vs. 12 months, aRR: 0.67, 0.51−0.87; 36 months, aRR: 0.60, 0.45−0.80; 48 months, aRR: 0.48, 0.35−0.66) and master's degree or higher education (vs. ≤ high school, aRR: 0.36, 0.17−0.66) were independently associated with lower seroconversion risk. Compared to non‐PrEP users in the past 2 years without a current clinical indication, those discontinuing PrEP had higher seroconversion risk, either with a clinical indication (aRR: 3.23, 1.74−6.46) or lack thereof (aRR: 4.30, 1.85−9.88). However, initiating (aRR: 0.14, 0.04−0.39) or persistently using PrEP (aRR: 0.33, 0.14−0.74) greatly lowered this risk. The risk for clinically indicated non‐PrEP users (aRR: 1.69, 0.98−3.22) remained positively associated with seroconversion but became statistically non‐significant (*p* = 0.079). These results remained robust in other analyses, including unweighted multivariable analyses (see Tables S1−S3).

## 4 DISCUSSION

In this cohort of HIV‐vulnerable SGM individuals, we observed high HIV incidence over 4 years of follow‐up (*n* = 303, 1.64 cases per 100 person‐years). Among those who initiated PrEP at some point during follow‐up, we observed 20 HIV seroconversions (0.41 per 100 person‐years)—a rate that was substantially lower than the overall cohort and substantially lower than those who did not initiate PrEP. Indeed, non‐PrEP users had 5.10 times higher rate of HIV seroconversion or PrEP use was associated with an 80% reduction in HIV incidence. Thus, PrEP use was a major factor in preventing HIV seroconversion. This finding highlights the critical need for enhanced interventions targeted at improved PrEP uptake, as well as continued efforts to address the structural barriers that make continued PrEP use difficult.

In multivariable modelling, several noteworthy factors were independently associated with HIV seroconversion: (1) *time*; (2) *race and ethnicity*; (3) *education* and *housing instability*; (4) *PrEP's usage clinical criteria*; and (5) *methamphetamine use*. We suggest that these factors lend insight into tailoring PrEP interventions by identifying those most at risk for seroconversion with greater precision while taking into consideration structural factors associated with HIV seroconversion risk.

First, *time*. We observed higher rates of HIV seroconversion early in follow‐up and these decreased over time. This was likely a result of those being most vulnerable to HIV succumbing to that vulnerability early during follow‐up. This highlights the urgency of providing means of prevention for those most vulnerable to HIV as soon as possible. Second, *race and ethnicity*. As is the case with observed HIV acquisitions among SGM individuals in the United States, racial/ethnic disparities evinced themselves in our cohort; even after adjusting for clinical indication for PrEP, non‐Hispanic Black (aRR 2.44) and Hispanic/Latinx (aRR 1.53) participants were significantly more likely to seroconvert than non‐Hispanic White participants. Such findings are not novel but underscore that HIV prevention resources must identify and prioritize successful PrEP implementation strategies for reaching and engaging Black and Latinx SGM individuals—and interventions themselves should not only target individual‐level factors, but also structural (racism) and intersecting stigmas (intersectionality), as well as concentrated HIV prevalence within social/sexual networks.

Third, *education* and *housing instability*. These factors evidenced themselves as independent predictors of HIV seroconversion in ways known in epidemiologic research to expose someone to a negative health outcome (greater education ameliorates risk, housing instability increases it). Identifying those experiencing unstable housing can help tailor intervention efforts and may offer an area for expanded collaboration between existing housing support and PrEP programming. Indeed, some clinical programmes have already adopted a model of care that integrates HIV prevention services (PrEP) with addressing material needs like housing, food insecurity and insurance navigation (e.g. Howard Brown Health).

Fourth, *PrEP's clinical indication criteria*. As noted, taking PrEP greatly reduced the risk of HIV seroconversion. Our findings were able to examine the joint associations between being clinically indicated for PrEP treatment and PrEP utilization; however, importantly, discontinuing PrEP, regardless of clinical indication, was associated with a higher risk of HIV seroconversion. Prior studies have likewise found higher seroconversion risk following gaps in PrEP use [28−30]. These findings highlight the importance of PrEP persistence and underscore that those who discontinued PrEP and may no longer be clinically indicated for PrEP might still benefit from monitoring for PrEP care. Equally, using the clinical criteria as a bar for *re‐entry* into PrEP care among former PrEP users may be misaligned. That is, knowing someone was a former PrEP user might be enough for re‐entry into care. These findings lend evidence for the potential relaxing of PrEP clinical criteria, though further research in this area is needed. Our study aligned with the 2017 CDC clinical criteria (which were in effect for most of this study). Whereas the CDC revised guidelines somewhat in 2021 to indicate discussing PrEP with all patients and to offer PrEP to those who had any risk of HIV acquisition [31].

Lastly, *methamphetamine*. Having used methamphetamine in the past year increased the risk of HIV seroconversion by nearly four‐fold (aRR 3.82). Aside from discontinuing PrEP and not being clinically indicated for PrEP (aRR 4.30), past year methamphetamine use had the second highest risk ratio for HIV seroconversion. In fact, methamphetamine was reported in the 12 months *prior* to seroconversion in 42% of cases. Although we lack the granularity to say methamphetamine *led* to a seroconversion, we can say that interventions targeted to those who use methamphetamine have the potential to identify individuals highly vulnerable to HIV. Recently, we have seen renewed attention to addressing increases in methamphetamine use and the direct role it plays in impeding our ability to make durable progress to end the HIV epidemic. Interventions that reduce methamphetamine use may also have the potential to impact HIV for SGM. To that end, interventions that reach SGM individuals who use methamphetamine may also provide an access point for PrEP education and intervention for those who would benefit most from PrEP's protection.

### 4.1 Limitations

Although participants were geographically diverse, our cohort is not a nationally representative sample. It was, however, intended to be a sample of individuals vulnerable to HIV. Certainly, many individuals clicked our link to screen for the study but did not complete the survey—most closing their browsers immediately. We do not have data on these individuals and cannot attest to their demographic/behavioural characteristics, and whether selection bias could have occurred. The study was, by design, intended to be more “hands off” (i.e. limited interaction) such that participants were assessed annually and via an online survey. This limited some granularity in our data; however, more frequent assessments have the potential to introduce bias through participant self‐monitoring of responses.

At‐home HIV test kits were sent to participants after they completed the surveys. This approach might have led to a conservative estimate of the HIV incidence rate, as non‐completers were not tested. However, any underestimation is likely minor due to the high survey completion rates. For most of the study, we used the OraSure HIV‐1 kit for HIV serology, which is an oral fluid sample. Collecting an oral fluid sample is less invasive than dried blood spot or whole blood; however, this device is a third‐generation HIV test. It is possible that a more sensitive test could have detected a greater number of HIV cases. However, any HIV acquisition missed at one time point would likely have been caught by the following. And although our study used some biomarkers, much of our data are self‐reported. Online surveys reduce social desirability bias, but we did collect data on sensitive topics (e.g. drug use) and thus it is possible some data could be underreported.

## 5 CONCLUSIONS

The *Together 5000* study observed high vulnerability to HIV acquisition and low PrEP uptake among a community‐based prospective cohort of SGM individuals who have sex with men. Those having adopted PrEP were at the lowest risk for acquiring HIV over the course of the study—meanwhile, those having adopted PrEP who later discontinued it demonstrated exceptional vulnerability to subsequent HIV seroconversion. Thus, not only are interventions necessary to engage individuals into PrEP care, but also retain them, and re‐engage those who may fall out of PrEP care. Inventions may increase their impact by prioritizing Black/Latinx individuals, those experiencing housing instability, with less education and those who use methamphetamine.

## COMPETING INTERESTS

DN received consulting fees from Abbvie and Gilead, and a grant from Pfizer to his institution. The authors report no competing interests.

## AUTHORS’ CONTRIBUTIONS

Study conception and design (CG, DAW, ABD, CM, MD, VVP, SAG, DRH and DN), data collection (CG, ABD, CM and MD), analysis and interpretation of results (CG, YG, DAW and ABD), draft manuscript preparation (CG, YG, DAW, ABD, CM, MD, PC, MR, DP, AWC, VVP, SAG, SH, DRH and DN). All authors reviewed the results and approved the final version of the manuscript.

## FUNDING

*Together 5000* (T5K) was funded by the National Institutes of Health (UH3 AI 133675 ‐ PI Grov). Other forms of support include the CUNY Institute for Implementation Science in Population Health, the Einstein, Rockefeller, CUNY Center for AIDS Research (ERC CFAR, P30 AI124414). DAW was supported, in part, by a career development award (K01 AA 029047).

## DISCLAIMER

While the NIH financially supported this research, the content is the responsibility of the authors and does not necessarily reflect the official views of the NIH.

## Supporting information


**Table S1**. Multivariable, unweighted discrete‐time survival analysis results for HIV seroconversion among sexual and gender minority individuals in a U.S.‐based longitudinal cohort, *Together 5,000*, 2017–2022.
**Table S2**. Multivariable, observation‐weighted discrete‐time survival analysis results with only significant factors (excluding food insecurity and gender) for HIV seroconversion among sexual and gender minority individuals in a U.S.‐based longitudinal cohort, *Together 5,000*, 2017–2022.
**Table S3**. Results from a multivariable, observation‐weighted discrete‐time survival analysis for HIV seroconversion among sexual and gender minority individuals in the U.S.‐based longitudinal cohort, *Together 5,000*, from 2017‐2022, with age treated as a categorical variable.

## Data Availability

The data that support the findings of this study are available from the corresponding author upon reasonable request.
