# Supplementary material for: Factors associated with PrEP‐era HIV seroconversion in a 4‐year U.S. national cohort of n = 6059 sexual and gender minority individuals who have sex with men, 2017−2022
Source: J Int AIDS Soc. 2024 Jun 25;27(6):e26312. doi: 10.1002/jia2.26312 (PMC11197961; doi:10.1002/jia2.26312)
Supplement: Supplementary file 1 — Table S1. Multivariable, unweighted discrete‐time survival analysis results for HIV seroconversion among sexual and gender minority individuals in a U.S.‐based longitudinal cohort, Together 5,000, 2017–2022. Table S2. Multivariable, observation‐weighted discrete‐time survival analysis results with only significant factors (excluding food insecurity and gender) for HIV seroconversion among sexual and gender minority individuals in a U.S.‐based longitudinal cohort, Together 5,000, 2017–2022. Table S3. Results from a multivariable, observation‐weighted discrete‐time survival analysis for HIV seroconversion among sexual and gender minority individuals in the U.S.‐based longitudinal cohort, Together 5,000, from 2017‐2022, with age treated as a categorical variable. [file JIA2-27-e26312-s001.docx]

Supplemental materials

Table S1. Multivariable, unweighted discrete-time survival analysis results for HIV seroconversion among sexual and gender minority individuals in a U.S.-based longitudinal cohort, *Together 5,000*, 2017-2022.

| Predictors | Adjusted Risk Ratio | 95% CI | *P* value |
| --- | --- | --- | --- |
| Other follow-ups vs. 12 months |  |  |  |
| Time (24 months) | 0.66 | 0.47-0.90 | **0.009** |
| Time (36 months) | 0.57 | 0.39-0.80 | **0.002** |
| Time (48 months) | 0.44 | 0.29-0.65 | **<0.001** |
| ns(age) | 0.92 | 0.42-2.00 | 0.838 |
| Racial/ethnic minorities vs. non-Hispanic White |  |  |  |
| Non-Hispanic Black | 2.42 | 1.65-3.48 | **<0.001** |
| Hispanic/Latinx | 1.54 | 1.14-2.08 | **0.005** |
| Other | 1.26 | 0.82-1.88 | 0.279 |
| Higher education vs. ≤ high school |  |  |  |
| Some college  Bachelor’s degree  Master’s degree or higher | 0.98  0.82  0.33 | 0.71-1.37  0.55-1.22  0.14-0.66 | 0.898  0.324  **0.004** |
| Unstable housing vs. stable housing | 1.66 | 1.22-2.24 | **0.001** |
| Meth use vs. non-meth use in past year | 4.00 | 2.69-5.92 | **<0.001** |
| Meth use vs. non-meth use 1-2 years ago | 1.31 | 0.88-1.95 | 0.190 |
| PrEP use patterns in past two years vs. No PrEP and not indicated for PrEP in past two years |  |  |  |
| No PrEP and indicated for PrEP | 1.69 | 0.88-3.77 | 0.155 |
| Initiated PrEP this year | 0.14 | 0.03-0.49 | **0.007** |
| Quit PrEP, not indicated for PrEP | 4.05 | 1.41-11.33 | **0.007** |
| Quit PrEP, indicated for PrEP | 2.99 | 1.42-7.09 | **0.004** |
| Persistent PrEP use | 0.27 | 0.10-0.77 | **0.013** |

Note: CI refers to confidence interval; ns refers to natural spline and PrEP to pre-exposure prophylaxis. 16,739 person-years were included in the analysis. The results were weighted with inverse probability of censoring weighting.

Table S2. Multivariable, observation-weighted discrete-time survival analysis results with only significant factors (excluding food insecurity and gender) for HIV seroconversion among sexual and gender minority individuals in a U.S.-based longitudinal cohort, *Together 5,000*, 2017-2022.

| Predictors | Adjusted Risk Ratio | 95% CI | *P* value |
| --- | --- | --- | --- |
| Other follow-ups vs. 12 months |  |  |  |
| Time (24 months) | 0.66 | 0.51-0.87 | **0.003** |
| Time (36 months) | 0.58 | 0.44-0.78 | **<0.001** |
| Time (48 months) | 0.47 | 0.34-0.64 | **<0.001** |
| ns(age) | 0.80 | 0.41-1.54 | 0.512 |
| Racial/ethnic minorities vs. non-Hispanic White |  |  |  |
| Non-Hispanic Black | 2.43 | 1.79-3.26 | **<0.001** |
| Hispanic/Latinx | 1.55 | 1.20-1.99 | **0.001** |
| Other | 1.15 | 0.80-1.62 | 0.436 |
| Higher education vs. ≤ high school  Some college  Bachelor’s degree  Master’s degree or higher | 1.01  0.84  0.34 | 0.78-1.32  0.61-1.17  0.17-0.64 | 0.929  0.305  **0.002** |
| Unstable housing vs. stable housing | 1.60 | 1.25-2.04 | **<0.001** |
| Meth use vs. non-meth use in past year | 3.97 | 2.85-5.51 | **<0.001** |
| Meth use vs. non-meth use 1-2 years ago | 1.34 | 0.96-1.87 | 0.087 |
| PrEP use patterns in past two years vs. no PrEP and not indicated for PrEP in past two years |  |  |  |
| No PrEP and indicated for PrEP | 1.74 | 1.01-3.31 | 0.063 |
| Initiated PrEP this year | 0.14 | 0.04-0.40 | **0.001** |
| Quit PrEP, not indicated for PrEP | 4.41 | 1.90-10.15 | **<0.001** |
| Quit PrEP, indicated for PrEP | 3.27 | 1.77-6.54 | **<0.001** |
| Persistent PrEP use | 0.33 | 0.15-0.76 | **0.008** |

Note: CI refers to confidence interval; ns refers to natural spline and PrEP to pre-exposure prophylaxis. 16,739 person-years were included in the analysis. The results were weighted with inverse probability of censoring weighting. Food insecurity variable which was significant in bivariate analysis but not in multivariable analysis was excluded in this model. Gender, not statistically significant in bivariate analysis, was not included in the model either.

Table S3. Results from a multivariable, observation-weighted discrete-time survival analysis for HIV seroconversion among sexual and gender minority individuals in the U.S.-based longitudinal cohort, *Together 5,000*, from 2017-2022, with age treated as a categorical variable.

| Predictors | Adjusted Risk Ratio | 95% CI | *P* value | |
| --- | --- | --- | --- | --- |
| Other follow-ups vs. 12 months |  |  |  | |
| Time (24 months) | 0.67 | 0.51-0.88 | **0.004** | |
| Time (36 months) | 0.61 | 0.45-0.81 | **0.001** | |
| Time (48 months) | 0.49 | 0.35-0.67 | **<0.001** | |
| Older age groups vs. 17~24 years |  |  |  | |
| 25~34 years | 0.83 | 0.61-1.13 | 0.219 |  |
| 35~44 years | 0.71 | 0.50-1.02 | 0.060 |  |
| 45~54 years | 0.74 | 0.46-1.16 | 0.201 |  |
| Racial/ethnic minorities vs. non-Hispanic White |  |  |  | |
| Non-Hispanic Black | 2.40 | 1.77-3.23 | **<0.001** | |
| Hispanic/Latinx | 1.50 | 1.16-1.93 | **0.002** | |
| Other | 1.10 | 0.76-1.56 | 0.604 | |
| *Cisgender male vs. other* | 0.91 | 0.54-1.69 | 0.742 |  |
| Higher education vs. ≤ high school  Some college  Bachelor’s degree  Master’s degree or higher | 1.05  0.89  0.37 | 0.81-1.37  0.64-1.25  0.18-0.69 | 0.743  0.504  **0.003** | |
| *Food insecure vs. food secure* | 1.00 | 0.80-1.26 | 0.978 |  |
| Unstable housing vs. stable housing | 1.59 | 1.22-2.05 | **<0.001** |  |
| Meth use vs. non-meth use in past year | 3.85 | 2.76-5.37 | **<0.001** | |
| Meth use vs. non-meth use 1-2 years ago | 1.42 | 1.01-1.99 | **0.042** | |
| PrEP use patterns in past two years vs. no PrEP and not indicated for PrEP in past two years |  |  |  | |
| No PrEP and indicated for PrEP | 1.74 | 1.01-3.31 | 0.063 | |
| Initiated PrEP this year | 0.14 | 0.04-0.40 | **0.001** | |
| Quit PrEP, not indicated for PrEP | 4.41 | 1.90-10.15 | **<0.001** | |
| Quit PrEP, indicated for PrEP | 3.27 | 1.77-6.54 | **<0.001** | |
| Persistent PrEP use | 0.33 | 0.15-0.76 | **0.008** | |

Note: CI refers to confidence interval, and PrEP to pre-exposure prophylaxis. 16,700 person-years were included in the analysis. The results were weighted with inverse probability of censoring weighting.
